# Loss of *AMPKα2* Impairs Hedgehog-Driven Medulloblastoma Tumorigenesis

**DOI:** 10.3390/ijms19113287

**Published:** 2018-10-23

**Authors:** Honglai Zhang, Rork Kuick, Sung-Soo Park, Claire Peabody, Justin Yoon, Ester Calvo Fernández, Junying Wang, Dafydd Thomas, Benoit Viollet, Ken Inoki, Sandra Camelo-Piragua, Jean-François Rual

**Affiliations:** 1 Department of Pathology, University of Michigan Medical School, Ann Arbor, MI 48109, USA; honglaiz@med.umich.edu (H.Z.); parksung@med.umich.edu (S.-S.P.); peabodyc@umich.edu (C.P.); jxy673@case.edu (J.Y.); esfernan@med.umich.edu (E.C.F.); thomasda@med.umich.edu (D.T.); 2 Department of Biostatistics, School of Public Health, University of Michigan, Ann Arbor, MI 48109, USA; rork@umich.edu; 3 Life Sciences Institute, University of Michigan, Ann Arbor, MI 48109, USA; wangjoy03@gmail.com (J.W.); inokik@umich.edu (K.I.); 4 Inserm, U1016, Institut Cochin, 75014 Paris, France; benoit.viollet@inserm.fr; 5 CNRS, UMR8104, 75014 Paris, France; 6 Université Paris Descartes, Sorbonne Paris cité, 75014 Paris, France

**Keywords:** medulloblastoma, sonic hedgehog, AMPK

## Abstract

The AMP-activated protein kinase (AMPK) is a sensor of cellular energy status that has a dual role in cancer, i.e., pro- or anti-tumorigenic, depending on the context. In medulloblastoma, the most frequent malignant pediatric brain tumor, several in vitro studies previously showed that AMPK suppresses tumor cell growth. The role of AMPK in this disease context remains to be tested in vivo. Here, we investigate loss of *AMPKα2* in a genetically engineered mouse model of sonic hedgehog (SHH)-medulloblastoma. In contrast to previous reports, our study reveals that *AMPKα2* KO impairs SHH medulloblastoma tumorigenesis. Moreover, we performed complementary molecular and genomic analyses that support the hypothesis of a pro-tumorigenic SHH/AMPK/CNBP axis in medulloblastoma. In conclusion, our observations further underline the context-dependent role of AMPK in cancer, and caution is warranted for the previously proposed hypothesis that AMPK agonists may have therapeutic benefits in medulloblastoma patients. Note: an abstract describing the project was previously submitted to the American Society for Investigative Pathology PISA 2018 conference and appears in *The American Journal of Pathology* (Volume 188, Issue 10, October 2018, Page 2433).

## 1. Introduction

Medulloblastoma is an embryonal tumor of the cerebellum and is the most common malignant brain tumor of childhood [1,2]. Genomics applied to medulloblastoma defined four medulloblastoma subgroups, each characterized by a distinct molecular/genetic signature, distinct patient demographics, and a distinct clinical profile (WNT, Sonic Hedgehog or SHH, Groups #3 and #4) [3,4]. More recently, integrative genomic analyses further underscored the highly heterogeneous and complex nature of medulloblastoma with a large spectrum of molecularly distinct consensus subgroups and subtypes within them [5,6,7]. Current therapeutic approaches to medulloblastoma are based on surgery, radiation, and non-targeted chemotherapy and are indistinguishably applied to all medulloblastoma subgroups. These therapies have led to significant improvements, with a 73% survival rate [1], but these results are achieved at a high cost to quality of life, e.g., neurocognitive or hormonal deficiencies [8,9]. Alternative therapeutic approaches are needed.

The AMP-activated protein kinase (AMPK) is a crucial energy sensor that controls cell metabolism and growth in response to low energy levels by phosphorylating a variety of substrates [10]. AMPK forms a heterotrimeric protein complex composed of three subunits that exist as multiple isoforms: a catalytic subunit (α1 or α2), a scaffolding subunit (β1 or β2), and an AMP-sensing subunit (γ1, γ2, or γ3) [10]. AMPK is allosterically activated by an increased AMP/ATP ratio when cells are metabolically starved. Upon metabolic stress, AMPK activation results in both the inhibition of anabolic, energy-consuming mechanisms (e.g., biosynthetic pathways and the cell cycle) and the activation of catabolic processes (e.g., the promotion of mitochondrial biogenesis and glycolysis) [10]. As the master metabolic guardian, AMPK modulates numerous targets, including both oncogenes and tumor suppressors [11,12]. Thus, AMPK has a dual role in cancer, tumor-suppressive, or pro-oncogenic, depending on the cellular or tissue context [13,14,15]. For example, as an inhibitor of the cell cycle and as a target of the LKB1 kinase [16,17,18], AMPK has long been considered an essential mediator of LKB1’s tumor-suppressive effect in cancer [13,19]. The tumor suppressor role of AMPK is also exemplified by the observation that inactivation of *AMPKα1* in murine B-cell lineages promotes *Myc*-driven lymphomagenesis [20] and that *AMPKα2* suppresses murine embryonic fibroblast transformation and tumorigenesis [21]. Yet, loss of *AMPKα* impairs tumor growth in vivo in various carcinogenic contexts [22,23,24,25,26,27]. Indeed, maintenance of the metabolic balance by AMPK is likely a critical process for survival during the metabolic stress that can occur in the tumor microenvironment [13,14,26].

In SHH-driven medulloblastoma tumorigenesis, several studies support a tumor-suppressive role for AMPK [28,29,30,31,32,33]. First, in medulloblastoma cells, AMPK phosphorylates the SHH pathway transcription factor GLI1, promoting its proteasomal degradation, thus inhibiting SHH signaling and SHH-driven medulloblastoma [28,29,30,33]. Second, a potential tumor-suppressive role for AMPK in SHH-driven medulloblastoma was also inferred by the fact that the increased survival observed upon *Hk2* KO in a mouse model of medulloblastoma is accompanied by a gain of AMPK activity [31,32]. In light of these observations, AMPK agonists could have therapeutic value for the treatment of SHH medulloblastoma patients. As noted above, though, the multifaceted role of AMPK in cancer warrants caution [13,14,15]. As a matter of fact, D’amico et al. recently proposed that a non-canonical SHH/AMPK axis promotes medulloblastoma via activation of CCHC type nucleic acid binding protein (CNBP), ornithine decarboxylase 1 (ODC1), and polyamine metabolism [34]. Though indirectly inferred, this study supports a putative pro-tumorigenic role for AMPK in SHH medulloblastoma [34]. Importantly, in all of the aforementioned studies, the role of AMPK in medulloblastoma was only investigated in vitro in medulloblastoma cells.

The potential role of AMPK as a promoter or as a suppressor of medulloblastoma tumorigenesis warrants further investigation and remains to be tested in vivo. Here, we describe the analysis of loss of *AMPKα2* in a genetically engineered mouse model of SHH-driven medulloblastoma. Remarkably, in disagreement with the previous studies supporting a tumor-suppressive role for AMPK in medulloblastoma tumorigenesis [28,29,30,31,32,33], our analysis reveals that loss of *AMPKα2* impairs SHH-driven medulloblastoma tumorigenesis.

## 2. Results

### 2.1. AMPKα2 Is Required for SHH-Driven Medulloblastoma In Vivo

**Figure 1:**
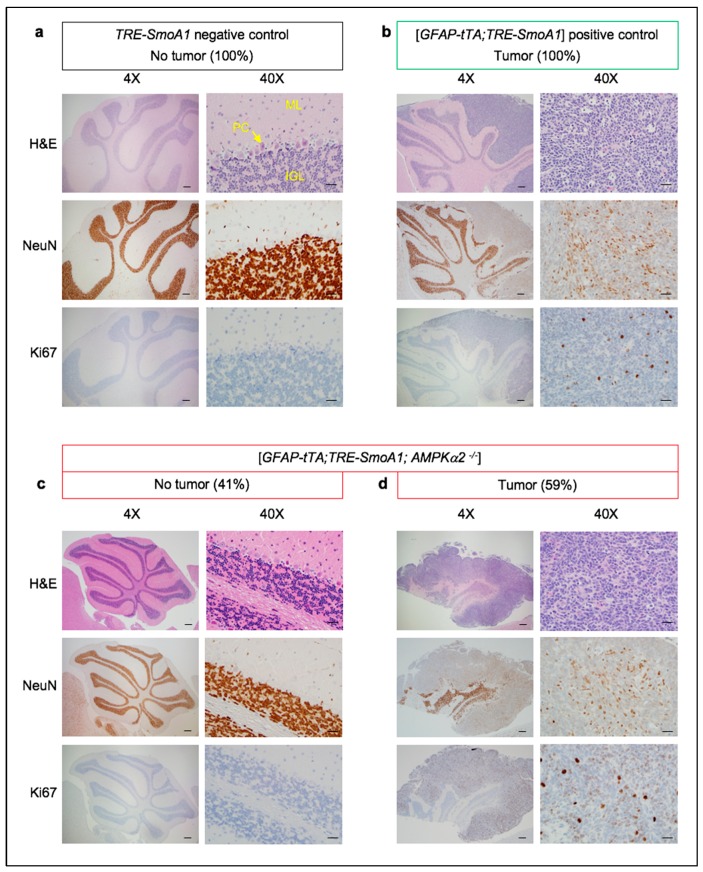
Loss of *AMPKα* impairs SHH medulloblastoma tumorigenesis in vivo. Histopathological examination of the cerebellums of (**a**) a Postnatal Day 63 (P63) *TRE-SmoA1* mouse, (**b**) a P56 [*GFAP-tTA;TRE-SmoA1*] mouse, (**c**) a one-year-old [*GFAP-tTA;TRE-SmoA1;AMPKα2^−/−^*] mouse representative of the 7/17 (41%) [*GFAP-tTA;TRE-SmoA1;AMPKα2^−/−^*] mice that do not show any evidence of tumor upon histological analysis, and (**d**) a P30 [*GFAP-tTA;TRE-SmoA1;AMPKα2^−/−^*] mouse representative of the 10/17 (59%) [*GFAP-tTA;TRE-SmoA1;AMPKα2^−/−^*] mice that show a tumor. The histological analysis includes H&E staining as well as Ki67 (marker of proliferation) and NeuN (marker of neuronal differentiation) immunohistochemistry (magnification: 4× and 40×). The different cell layers of the cerebellum, i.e., the molecular (ML), Purkinje cell (PC), and internal granular cell (IGL) layers are labeled in the 40× *TRE-SmoA1* control H&E picture. Scale bars: 200 μm (4×) or 25 μm (40×).

**Figure 2:**
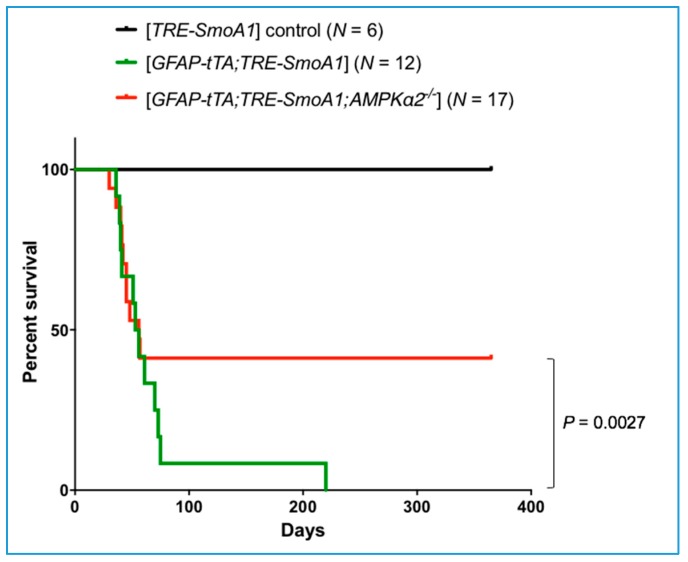
Loss of *AMPKα* increases survival in *SmoA1* mouse model of SHH medulloblastoma. Survival analysis of *AMPKα2* KO in *SmoA1*-driven medulloblastoma. Three groups of *C57BL/6* mice were assessed for survival: (i) *TRE-SmoA1*, (ii) [*GFAP-tTA;TRE-SmoA1*], and (iii) [*GFAP-tTA;TRE-SmoA1;AMPKα2^−/−^*]. The *AMPKα2* KO mice have a significantly lower chance than the *AMPKα2* WT mice of developing medulloblastoma [59% (10/17) for *AMPKα2* KO versus 100% (12/12) for *AMPKα2* WT; *p* = 0.0027, likelihood ratio chi-square test]. For the log-rank test, which is designed to be most powerful when hazards are proportional, we obtained *p* = 0.108. Indeed, the *AMPKα2* KO mice that develop medulloblastoma succumb to the tumor at the same pace as WT mice in the first 60 days.

We investigated the role of *AMPKα2* in vivo in medulloblastoma using the [*GFAP-tTA;TRE-SmoA1*] genetically engineered mouse model of SHH-driven medulloblastoma [35,36] in combination with the *AMPKα2* KO mouse [37]. We note that, as previously described [37], *AMPKα2* KO mice are born with the expected Mendelian ratio, are fertile, appear indistinguishable from their WT littermates, and have a normal lifespan. Accordingly, *AMPKα2* KO mice exhibit normal cerebellar development with properly tri-laminated cerebellar cortex (molecular, Purkinje cell and internal granular cell layers). Three groups of C57BL/6 mice were analyzed: (a) *TRE-SmoA1* mice: the oncogenic gain-of-function allele of *Smo*, i.e., *SmoA1*, is not expressed in the absence of *GFAP-tTA* in these negative control group mice; (b) [*GFAP-tTA;TRE-SmoA1*] mice: all mice develop medulloblastoma in this positive control group; (c) [*GFAP-tTA;TRE-SmoA1;AMPKα2^−/−^*] mice: this test group allows us to assess the extent to which *AMPKα2* KO modulates tumor incidence. Genotyping data for each group is shown in Figure S1. For each study group, we performed histological (Figure 1) and survival (Figure 2) analyses.

Cerebellar development is normal in the *TRE-SmoA1* control mice. Indeed, as expected for adult mice, we observe properly tri-laminated molecular, Purkinje cell, and internal granular cell layers, but no external granular cell layer. The strong staining for the neuronal marker NeuN indicates the presence of normal cerebellar neurons in the internal granular layer. The absence of staining for Ki-67 indicates that cells are in a quiescent, non-proliferative stage (Figure 1a). As previously described [35,36], all [*GFAP-tTA;TRE-SMOA1*] mice develop large medulloblastoma tumors that can extend along the entire rostral-caudal length of the cerebellum, account for more than a quarter of the total cerebellar volume, express NeuN, indicative of neuronal origin, and show marked proliferative activity (positive for Ki67) (Figure 1b). The vast majority (11/12) of the [*GFAP-tTA;TRE-SMOA1*] mice succumb to the tumor within 80 days (Figure 2), as previously described [35,36]. One [*GFAP-tTA;TRE-SMOA1*] mouse survived beyond 200 days with a very large tumor mass identified post-mortem (Figure S2). We note that, apart from medulloblastoma formation, no other types of tumors can be detected in the brain or the other organs of [*GFAP-tTA;TRE-SMOA1*] mice.

In contrast, 41% (7/17) of the [*GFAP-tTA;TRE-SmoA1;AMPKα2^−/−^*] mice survived beyond one year (Figure 2) and do not show any evidence of tumor upon histological analysis (Figure 1c). Two of these seven mice show focal, microscopic remnants of external granular cell (EGC) neurons in groups no larger than 10 cells, which were non-proliferative and non-neoplastic (Figure S3). These cells likely correspond to granular cell neurons that have failed to properly migrate into the internal granular layer, a process that is dependent on SHH signaling inhibition [38]. Thus, the *AMPKα2* KO mice have a significantly lower chance of developing medulloblastoma compared to the *AMPKα2* WT mice [59% (10/17) for *AMPKα2* KO versus 100% (12/12) for *AMPKα2* WT; *p* = 0.0027]. Our results demonstrate the pro-tumorigenic role of *AMPKα2* in medulloblastoma. We note that the number of pyknotic cells undergoing apoptosis in either the normal cerebellar tissues (very rare occurrence, <1%) or the tumorigenic tissues (~10–20%) remains the same in the *AMPKα2* WT and KO mice. Thus, the effect of *AMPKα2* on apoptosis is not a likely to be a major factor underlying the pro-tumorigenic role of *AMPKα2* in medulloblastoma. Below, we explore the potential effect of *AMPKα2* KO on CNBP.

### 2.2. Loss of AMPKα2 Results in the Decreased Expression of the CNBP Protein

**Figure 3:**
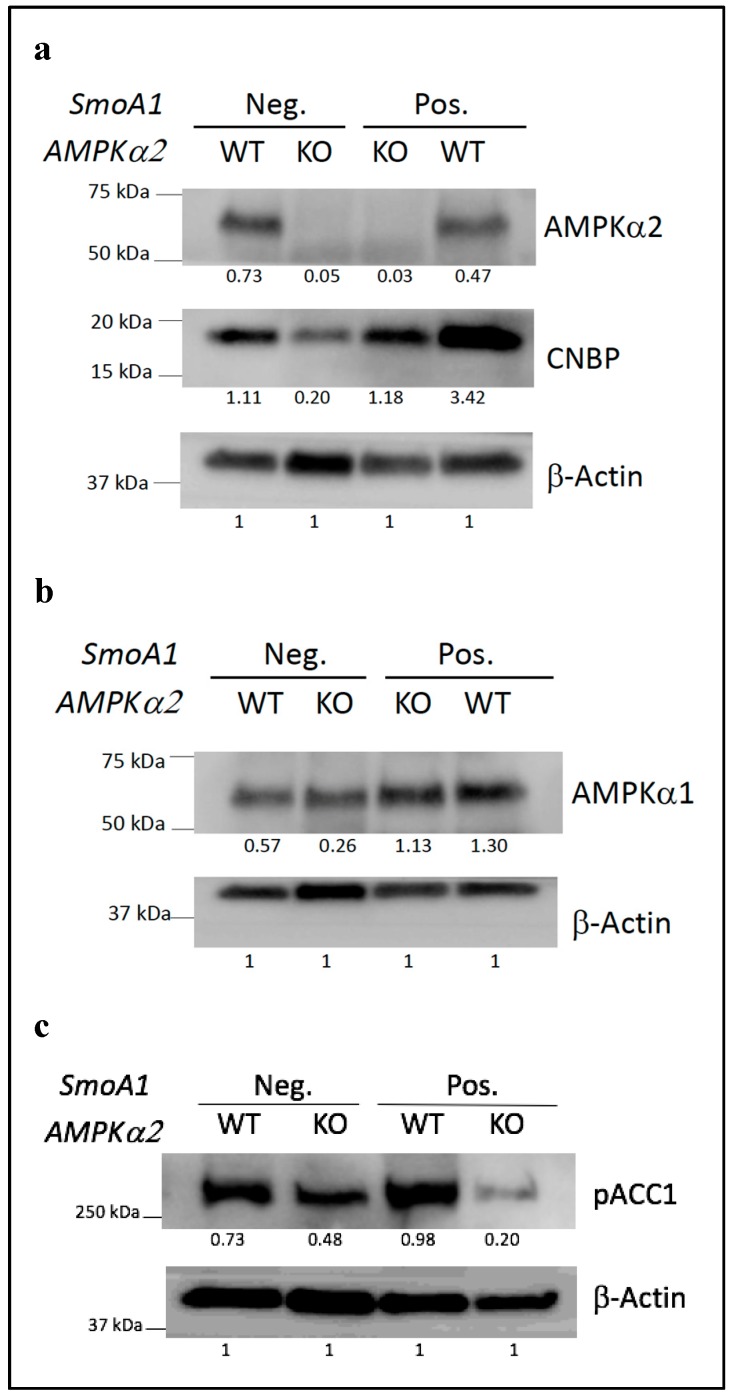
Loss of *AMPKα2* results in lower level of expression of the CNBP and pACC1 proteins in both physiological (*SmoA1−*) and pathophysiological (*SmoA1+*) contexts. Western blot analyses of the AMPKα2 (**a**), AMPKα1 (**b**), CNBP (**a**), pACC1 (**c**), and β-Actin proteins in whole cerebellum protein extracts obtained from WT, *AMPKα2^−/−^*, [*GFAP-tTA;TRE-SmoA1; AMPKα2^−/−^*], and [*GFAP-tTA;TRE-SmoA1*] Postnatal Day 16 (P16) littermate mice. Below each Western blot band, we show the ratio of the band intensity of the targeted protein over the band intensity of β-Actin.

In response to SHH signaling, AMPK phosphorylates CNBP, thus promoting its interaction with SUFU and its stabilization [34]. Subsequently, the increased level of CNBP protein expression results in the increased translation of ODC1 and associated activation of polyamine metabolism, which is essential for the SHH-dependent proliferation of medulloblastoma cells [34]. In their study, D’amico et al. also showed that targeting CNBP or ODC1 impairs SHH-driven medulloblastoma and thus indirectly inferred that AMPK may be required for tumor formation in this context [34]. Our observation that *AMPKα2* KO impairs tumor progression in the *SmoA1* mouse model of medulloblastoma directly verifies this hypothesis (Figure 1 and Figure 2). To further assess the relevance of this SHH/AMPK/CNBP axis in SHH medulloblastoma, we investigated the level of protein expression of CNBP in both normal and tumorigenic cerebellums in the presence or in the absence of *AMPKα2*. We observed that, in both physiological (*SmoA1−*) and pathophysiological (*SmoA1+*) contexts, the loss of *AMPKα2* resulted in lower levels of the CNBP protein (Figure 3a). In conclusion, notwithstanding that other AMPK substrates [11,12] may also contribute to the pro-tumorigenic role of *AMPKα2* in medulloblastoma, these results further support the hypothesis of a critical role for the SHH/AMPK/CNBP axis in SHH-driven medulloblastoma.

Loss of *AMPKα2* can be associated with a compensatory increase in AMPKα1 protein, e.g., in the muscle [39]. We investigated this effect in the cerebellum. While the level of expression of AMPKα1 is higher in the *SmoA1+* mice than in the *SmoA1−* mice, it remains the same in the control and the *AMPKα2* KO mice in both the *SmoA1− and SmoA1+* contexts (Figure 3b). Thus, in both normal and tumorigenic cerebellums, the deficiency in AMPKα2 was not compensated for by AMPKα1 overexpression.

Similarly, we investigated the level of expression of phosphorylated acetyl-CoA carboxylase alpha (pACC1) in both normal and tumorigenic cerebellums in the presence or in the absence of *AMPKα2*. ACC1 is a key enzyme in the fatty acid synthesis pathway that mediates the conversion of acetyl-CoA to malonyl-CoA. ACC1 phosphorylation by AMPK results in its inactivation, thus inhibiting lipogenesis [40,41]. We observed that, in the *SmoA1+* context, the loss of *AMPKα2* resulted in lower levels of the pACC1 protein (Figure 3c). This analysis of pACC1 suggests a potential impact associated with *AMPKα2* KO on lipogenesis. Interestingly, while a pro-tumorigenic role of ACC1 has been previously described in various contexts [42], a tumor-suppressive role has also been suggested. Indeed, ACC1 inhibition by AMPK is required to maintain NADPH levels and in vivo growth of lung and breast tumor xenografts [23]. Similarly, impaired inhibition of ACC1 upon *AMPKα2* KO may result in low NADPH levels in medulloblastoma cells and thus may contribute to the pro-tumorigenic role of AMPKα2 in medulloblastoma.

### 2.3. Frequent Copy Number Gains for the AMPK, CNBP, and ODC1 Genes in SHH and Group #3 Medulloblastoma Patients

**Figure 4:**
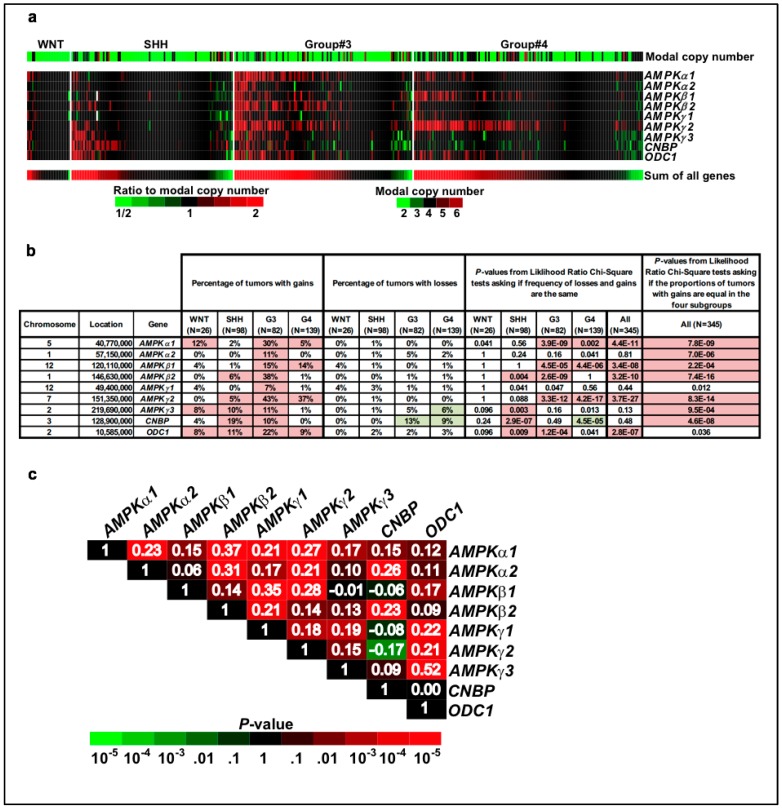
Frequent copy number gains for the AMPK, CNBP, and ODC1 genes in SHH and Group #3 medulloblastoma patients. (**a**) Estimated ratio of copy number to modal copy number for each tumor for 345 medulloblastomas, including 26 WNT, 98 SHH, 82 Group #3, and 139 Group #4 samples. Modal copy number estimates are shown in the first row and the rows for individual genes show copy number estimates normalized to the modal copy number. The “sum of all genes” row was computed from the sum of the log-transformed ratios for the nine genes and was used to determine the sorting of samples in each group. (**b**) The percentage of tumors within each of the four subgroups with copy number gains [log2($\frac{copy\;number}{modal\;copy\;number}$) > 0.4] or losses [log2($\frac{copy\;number}{modal\;copy\;number}$) < −0.4]. Gains or losses in greater than 5% of the tumors in a given group are highlighted with red or green background, respectively. Similarly, *p* values < 0.01 are highlighted. (**c**) Analysis of the correlations of log-transformed normalized copy number estimates shows that positive correlations are frequent. Pearson correlations are shown as numbers in white, while their significance is indicated by the color of the background in each cell.

We analyzed the publicly available copy number data for 345 medulloblastoma samples [5] (Figure 4). Notably, *AMPK* genes do not have frequent copy number losses in medulloblastoma, the sole exception being the loss of *AMPKγ3* in 6% of Group #4 tumors. In contrast, *AMPK* genes tend to have frequent copy number gains in medulloblastoma in general, and particularly in Group #3 tumors, which have frequent gains for the *AMPKα1* (30%)*, AMPKβ2* (38%), and *AMPKγ2* (43%) genes. *AMPKα2* also has frequent copy number gains in Group #3 tumors (11%) (Figure 4a,b). These observations resonate with the previous report that Group #3 medulloblastoma has frequent chromosome 1 gains [43]. In SHH medulloblastoma patients, we observed frequent copy number gains for *AMPKβ2* (6%), *AMPKγ2* (5%) and *AMPKγ3* (10%). Interestingly, strong copy number gains are also observed for *CNBP* and *ODC1* in SHH medulloblastoma (19% and 11%, respectively), as well as in Group #3 medulloblastoma (10% and 22%, respectively). These observations draw yet another interesting parallel with the D’amico et al. study in that their immunohistochemistry analysis of CNBP and ODC1 in a cohort of 42 medulloblastoma patients revealed high levels of expression of both proteins in both SHH and Group #3 medulloblastoma [34]. The extent to which genetic events co-occur or not in patients can reveal functional relationships between genes, e.g., dependence or redundancy [44]. We found that the copy numbers for the nine genes under investigation are often positively correlated, with a remarkable 21 out of 33 non-syntenic pairs of genes having significant (*p* < 0.01) positive correlations (Figure 4c). In other words, not only are *AMPK*, *CNBP,* and *ODC1* genes frequently gained in medulloblastoma, but these gains tend to co-occur more often than expected at random, suggesting that the co-occurrence of these gains may provide a synergistic advantage. Together, these observations are in agreement with a pro-tumorigenic role for the SHH/AMPK/CNBP axis in medulloblastoma.

## 3. Discussion

In this study, we demonstrate that loss of AMPKα2 impairs SHH-driven medulloblastoma tumorigenesis. This is a surprising observation given the previous reports supporting a tumor-suppressive role for AMPK in medulloblastoma tumorigenesis [28,29,30,31,32,33] and the fact that the subunit AMPKα2 alone can suppress tumorigenesis in other contexts [21,45,46]. In fact, together with a previous analysis of AMPKα2 in mammary carcinoma [25], our analysis represents the first in vivo evidence that the loss of AMPKα2 alone can impair oncogenesis.

Several reasons may underlie this apparent AMPK paradox, both in cancer in general and in medulloblastoma in particular. As previously reviewed, AMPK modulates numerous targets both to inhibit anabolism and to promote catabolism. As such, AMPK has a dual role in cancer, tumor-suppressive or pro-tumorigenic, depending on the cellular or tissue context [13,14,15]. For example, on the one hand, as an inhibitor of the mTOR complex 1 and the cell cycle, AMPK can be tumor-suppressive [19,20]. On the other hand, maintenance of the metabolic balance by AMPK may be critical for survival during metabolic stress that can occur in the tumor microenvironment—hence the requirement for AMPK activity in some cancer cells [22,23,24,25,26,27]. Similarly, in medulloblastoma, the role of AMPK as both an inhibitor of GLI1 and SHH signaling [28,29,30,33] (tumor-suppressive effect) as well as a promoter of CNBP and polyamine metabolism (pro-tumorigenic effect) supposes a dual role for AMPK in this disease context. In agreement with the observation that CNBP protein levels are decreased in the absence of AMPKα2, we observed that the pro-tumorigenic function of AMPKα2 prevails in the context of the highly penetrant [GFAP-tTA;TRE-SMOA1] mouse model of medulloblastoma. It would be interesting, though, to assess the loss of AMPKα2 in other mouse models of medulloblastoma with lower incidence/longer latency [47,48], in order to investigate which of AMPKα2’s roles, tumor-suppressive or pro-tumorigenic, prevails in these contexts.

The apparent discrepancy between our results observed in mouse and the previously described tumor-suppressive role of AMPK in human medulloblastoma cell lines [28,29,30,33] could be due to the species-specific role of AMPK in the cerebellum. While all studies in which the role of AMPK has been directly tested genetically in human medulloblastoma cells agree, i.e., AMPK suppresses SHH-driven cell growth, the role of AMPK in mouse cell lines is debated [28,29,30,33]. For example, several studies by the Yang laboratory showed that AMPK phosphorylates GLI1 at multiple sites and targets it for proteasomal degradation in murine cells [28,29,33]. Yet, Di Magno et al. argue that AMPK phosphorylates GLI1 at the unique residue Ser408, which is conserved only in primates but not in other species [30]. If the regulation of GLI1 by AMPK does not occur in mouse, as suggested by Di Magno et al., this species-specific difference could explain why our observations obtained in mouse contradict the previous reports that AMPK suppresses growth in human SHH medulloblastoma [28,29,30,31,32,33]. Further investigation of the species-specific effect of AMPK on GLI1 and medulloblastoma is warranted.

In some contexts, AMPKα1 and AMPKα2 mediate specific, non-redundant functions, e.g., AMPKα2^−/−^ but not AMPKα1^−/−^, mice are resistant to hypoglycemic AICAR effects [37,49]. In other contexts, AMPKα1 and AMPKα2 are genetically redundant, e.g., while both AMPKα1 and AMPKα2 are dispensable for development, the combined loss of both genes is embryonic lethal at E10.5 [50]. While we cannot exclude the possibility that a complete KO of AMPKα may have an even more dramatic effect on medulloblastoma tumorigenesis and survival, our results suggest that AMPKα2 has a specific function in this context. This lack of genetic redundancy may underlie various mechanisms, as previously reviewed [51,52]. Could this be due to different biochemical function for AMPKα1- and AMPKα2-containing heterotrimeric complexes? AMPKα2 may be the target of different regulators and/or AMPKα2 may target different substrates. In that regard, it would be interesting to assess whether CNBP is specifically targeted by AMPKα2- but not by AMPKα1-containing heterotrimeric complexes. Could this be due to variations in the spatio-temporal expression for AMPKα1 and AMPKα2? Cell types may express one gene, but not the other. In agreement with an AMPKα2-specific function in SHH medulloblastoma and the fact that the cerebellar granular neuron progenitor cell (CGNP) is the “cell of origin” in SHH-driven medulloblastoma [53], AMPKα2 is expressed at higher level than AMPKα1 in cerebellar granule neurons [54].

Genomics applied to medulloblastoma defined four medulloblastoma subgroups (WNT, SHH, and Groups #3 and #4) [3,4] and, more recently, different subtypes within them [5,6,7]. D’amico et al. previously showed that targeting CNBP or ODC1 impairs SHH-driven medulloblastoma and inferred that the SHH/AMPK/CNBP axis may be pro-tumorigenic in this context [34]. Notwithstanding the debate on the nature of the effect (pro- versus anti-tumorigenic) [28,29,30,31,32,33,34], our observation that AMPKα2 impairs tumor progression in the SmoA1 mouse model of medulloblastoma validates this hypothesis. Is AMPK functionally relevant in other medulloblastoma subgroups? We observed high copy numbers for the AMPK, CNBP, and ODC1 genes not only in SHH but also in Group #3 medulloblastoma. Taken together with the complementary report that both SHH and Group #3 medulloblastomas show a high level of expression of CNBP and ODC1 proteins [34], the SHH/AMPK/CNBP axis could then have a critical role in both the SHH and Group #3 subgroups. Group #3 medulloblastomas account for ~25% of all medulloblastomas, are characterized by a transcriptional signature associated with photoreceptors and gamma aminobutyric acid–secreting (GABAergic) neurons, and are associated with a poor prognosis [3]. Though differences in the metabolic profiles between medulloblastoma subgroups have not yet been comprehensively examined, the higher degree of aggressiveness associated with Group #3 medulloblastomas may translate to a higher need for energy and thus a higher sensibility to the proper maintenance of the metabolic balance by AMPK under metabolic stress conditions. Investigation of AMPK KO in mouse models of Group #3 medulloblastoma, when they become available, is warranted.

Our study reveals a pro-tumorigenic role for AMPKα2 in SHH medulloblastoma. In light of the previous reports on the tumor-suppressive role of AMPK in SHH medulloblastoma, this observation further underscores the multifaceted role of AMPK in cancer. It also warrants caution to the previously proposed use of AMPK agonists for the treatment of cancer patients in general, and in medulloblastoma patients in particular.

## 4. Materials and Methods

### 4.1. [GFAP-tTA;TRE-SmoA1] Mouse Model

We studied medulloblastoma tumorigenesis in the absence or presence of AMPKα2 in the previously published bitransgenic [GFAP-tTA;TRE-SmoA1] model [35,36], where the expression of the tetracycline-regulated transactivator (tTA) is driven by a GFAP promoter and the expression of oncogenic SmoA1 is under the control of the tetracycline responsive element (TRE). The experimental breeders used in this study were an AMPKα2 KO mouse [37], a TRE-SmoA1 mouse [35], and a GFAP-tTA mouse [55], all of which were maintained on a C57/BL6 background for at least five generations prior to initiating experiments. TRE-SmoA1, [GFAP-tTA;TRE-SmoA1], and [GFAP-tTA;TRE-SmoA1;AMPKα2^−/−^] mouse littermates were generated by crossing [GFAP-tTA;AMPKα2^+/−^] mice with [TRE-SmoA1;AMPKα2^+/−^] mice. Animals that meet the guidelines for end-stage illness and/or found to be at protocol endpoint (evidence of large tumor formation, enlarged dome head, and/or severe neurological dysfunction) were humanely euthanized in accordance with the institutional guidelines for the welfare of experimental animals. Maintenance of mouse colonies and experimental procedures were approved by the University of Michigan Committee on the Use and Care of Animals.

### 4.2. Mouse Genotyping

The mouse genotyping experiments were performed as previously described [36], with minor modifications. Genotyping experiments were performed by PCR analysis using tail genomic DNA obtained from pups at Postnatal Days 10 (P10) and 14 (P14). Genotyping of *AMPKα2* was determined by PCR using the following oligonucleotides: forward: 5′-gcttagcacgttaccctggat-3′ and WT reverse: 5′-gtcttcactgaaatacatagca-3′ or mutant reverse: 5′-gcattgaaccacagtccttcctc-3′. Genotyping of the *GFAP-tTA* transgene was determined by PCR using the following oligonucleotides: forward: 5′-ctcgcccagaagctaggtgt-3′ and reverse: 5′-ccatcgcgatgacttagt-3′. Genotyping of the *TRE-SmoA1* transgene was determined by PCR using the following oligonucleotides: forward: 5′-ggaactgatgaatgggagca-3′ and reverse: 5′-gggaggtgtgggaggttt-3′. For internal control genotyping, we used the following primers (forward: 5’-caaatgttgctgtctggtg-3’ and reverse: 5’-gtcagtcgagtgcacagttt-3’).

### 4.3. Immunohistochemistry

Immunohistochemistry (IHC) experiments were performed as previously described [36].

### 4.4. Western Blot Analyses

Whole cerebellum protein samples were prepared using RIPA buffer, sonicated three times on ice, and supernatants were collected after centrifugation. The resulting cerebellum protein extracts were separated on acrylamide gels, transferred to PVDF membranes, and proteins were detected using standard immunoblotting techniques. The following antibodies were used: AMPKα1 (Bethyl Laboratory^®^, Montgomery, TX, USA; Cat# A300-507A), AMPKα2 (Bethyl Laboratory^®^, Cat# A300-508A), pACC1 (Cell Signaling^®^, Danvers, MA, USA; Cat# 11818), β-actin (Cell Signaling^®^, Cat# 5125), and goat α-rabbit IgG (Jackson Immunoresearch Laboratory^®^, West Grove, PA, USA; Cat# 111-035-045). The CNBP antibody [34] was a gift from Dr. Gianluca Canettieri, Sapienza University of Rome, Italy. The intensities of Western blot signal bands were quantified using Gel Analysis in ImageJ.

### 4.5. Copy Number Variations in Medulloblastoma Patients

We analyzed the publicly available copy number data for medulloblastoma samples estimated from short-read DNA sequencing [5]. We downloaded a single segment file holding estimated log_2_($\frac{copy\;number}{2}$) data from PedcBioportal (http://pedcbioportal.org/). Following the methods of the original paper [5], we excluded 16 samples and analyzed the remaining 345. Many tumors had chromosome doublings, so we normalized our data by first computing the average of the log_2_($\frac{copy\;number}{2}$) estimates for each chromosome and then counted how many chromosomes gave estimates within 0.1 unit of each possible log-transformed integer copy number. We then selected the most common integer chromosomal copy number for each tumor, after excluding those chromosomes with the most frequent copy number changes (Chr. 6, 7, 8, and 17, X and Y), and referred to this as the modal copy number. Among the 345 tumors, we observed 212 tumors with a 2-modal copy number, 19 tumors with a 3-modal copy number, 91 tumors with a 4-modal copy number, 2 tumors with a 5-modal copy number, and 21 tumors with a 6-modal copy number. Our final estimates of the relative gene copy numbers were of log_2_($\frac{copy\;number}{modal\;copy\;number}$).

## Supplementary Materials

Supplementary Materials can be found at http://www.mdpi.com/1422-0067/19/11/3287/s1.

## Abbreviations

AMPK	AMP-activated protein kinase
CGNPs	Cerebellar granular neuron progenitor cells
Chr.	Chromosome
DAB	Diaminobenzidine
GABAergic	Gamma aminobutyric acid–secreting
H&E	Hematoxylin and eosin
HRP	Horseradish peroxidase
IHC	Immunohistochemistry
IGL	Internal granular cell
ML	Molecular
P10	Postnatal day 10
pACC1	phosphorylated acetyl-CoA carboxylase alpha
PC	Purkinje cell
SHH	Sonic Hedgehog
TRE	Tetracycline responsive element
WT	Wild type
