# Supplementary material for: Loss of AMPKα2 Impairs Hedgehog-Driven Medulloblastoma Tumorigenesis
Source: Int J Mol Sci. 2018 Oct 23;19(11):3287. doi: 10.3390/ijms19113287 (PMC6274763; doi:10.3390/ijms19113287)

Supplementary Figure legends

Supplementary Figure S1. Mouse genotyping.

Shown in the figure is the genotype analysis of WT, *AMPKα2*$^{-/-}$, [*GFAP-tTA;TRE-SmoA1; AMPKα2*$^{-/-}$] and [*GFAP-tTA;TRE-SmoA1*] mice.

Supplementary Figure S2. Histopathological examination of a very large tumor in the [*GFAP-tTA;TRE-SMOA1*] mouse that survived beyond 200 days.

The histological analysis includes H&E staining as well as Ki67 (marker of proliferation) and NeuN (marker of neuronal differentiation) immunohistochemistry (magnification: 2X, 4X and 40X). Scale bars: 500 μm (2X), 200 μm (4X) or 25 μm (40X).

Supplementary Figure S3. Histopathological examination of non-proliferative, non-neoplastic microscopic remnants of granular cell neurons in a one-year-old [*GFAP-tTA;TRE-SMOA1;AMPKα2*$^{-/-}$] mouse.

The histological analysis includes H&E staining as well as Ki67 (marker of proliferation) and NeuN (marker of neuronal differentiation) immunohistochemistry (magnification: 4X and 40X). The different cell layers of the cerebellum, i.e. the molecular (ML), Purkinje cell (PC) and internal granular cell (IGL) layers are labeled in the 40X H&E picture. One example of remnant is labeled with a red arrow in the 40X pictures. Scale bars: 200 μm (4X) or 25 μm (40X).

Supplemetary Figure S1. (Zhang)

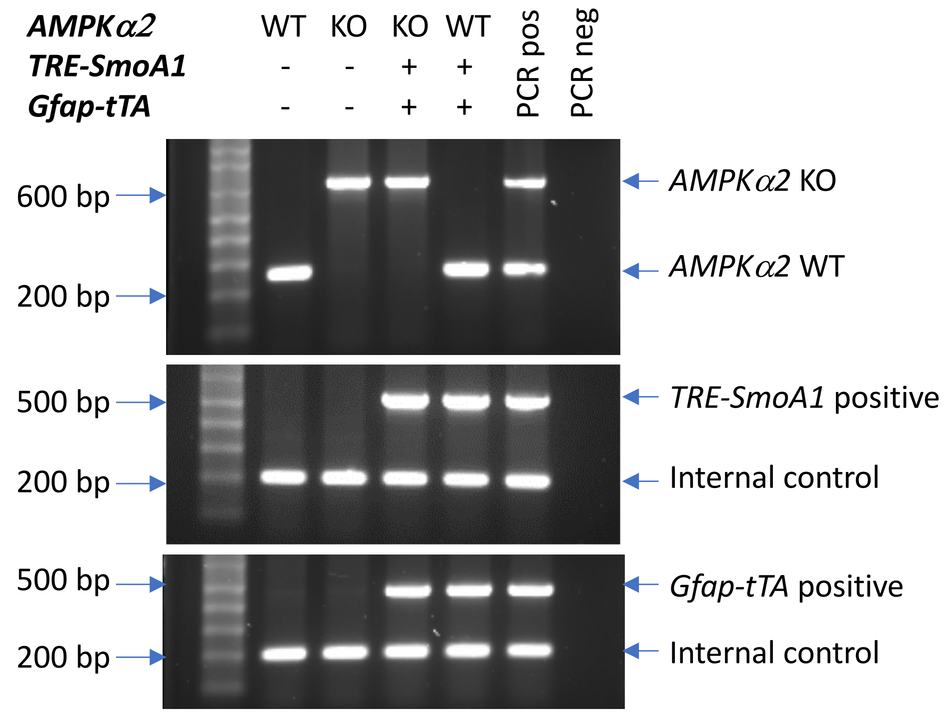

Supplemetary Figure S2. (Zhang)

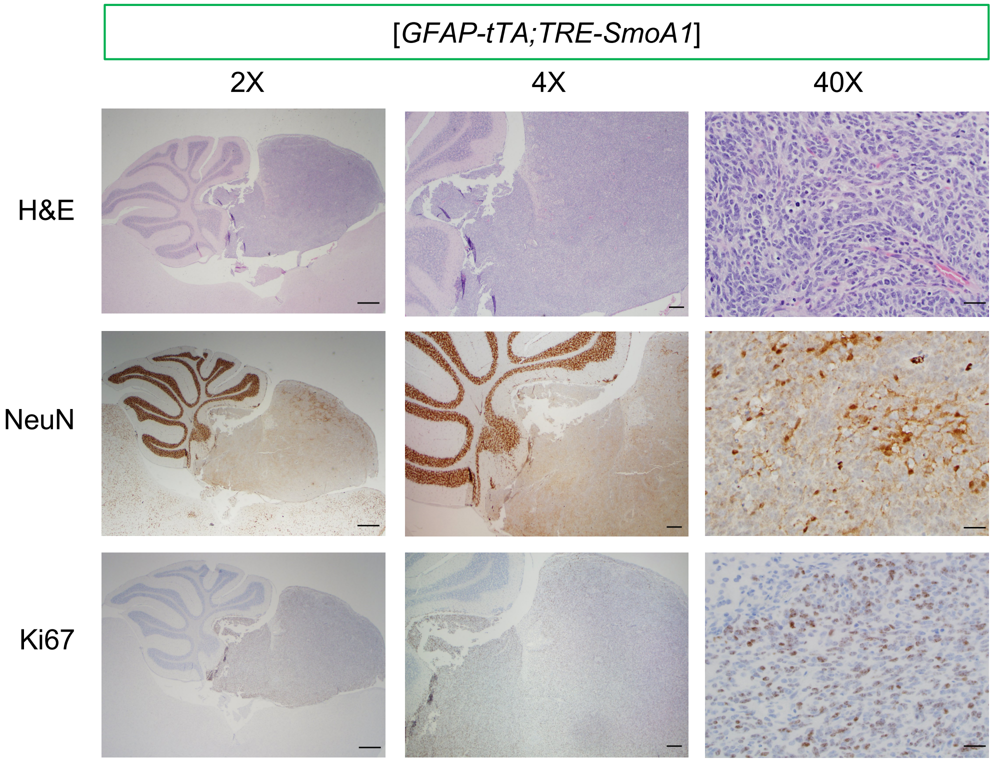

Supplemetary Figure S3. (Zhang)

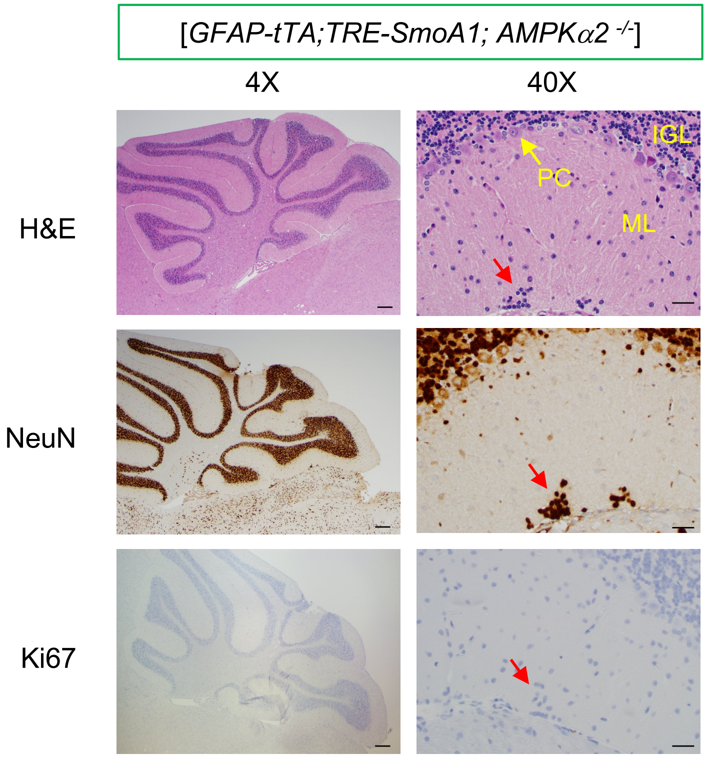

Supplement: Supplementary file 1 [file ijms-19-03287-s001.pdf]
